# Ph-Negative Chronic Myeloproliferative Neoplasm (Primary Myelofibrosis) – as One of the Reasons of the Budd-Chiari Syndrome

**DOI:** 10.4084/MJHID.2012.047

**Published:** 2012-06-30

**Authors:** M. Sokolova, N. Tsvetaeva, G. Sukhanova, S. Vasiliev, A. Misurin, M. Sanatko, M. Nareiko, V. Rudakova, E. Semenova, N. Khoroshko

**Affiliations:** Hematology Research Centre, Moscow, Russia

**Dear Editor,**

Valla and his team pioneered the thorough search for latent myeloproliferative neoplasms (MPN) in patients with splanchnic thrombosis.^1^ They noticed that “primary myeloproliferative disorder, often without peripheral blood changes, is a major cause of hepatic vein thrombosis”.^1^ Valla provided precious guidelines for anticoagulant therapy suggesting to start anticoagulation as early as possible, since it is much more effective in preventing extension to or from the portal vein than in inducing complete recanalization of extrahepatic portal vein obstruction.^2^,^3^

The problem of latent or evident MPN in young people with splanchnic venous thrombosis also was investigated by Teofili L, De Stefano V, Leone G.^4^ Special attention was paid to the problems of Adult Splanchnic Vein Thrombosis and clinical course of MPN [].^4^,^5^ The main conclusion of their research was that the leading cause of splanchnic vein thrombosis are MPN, which are diagnosed, even if a latent form, in 50% of patients with Budd-Chiari syndrome (BCS), furthermore deficiency of natural anticoagulants can favor the insurgence of BCS.^5^

Our case further shows that in absence of classical criteria and molecular diagnostic tools the diagnosis of MPN can be overlooked and the splanchnic thrombosis can be attributed in a young adult to other causes of thrombosis. We observed the insurgence of ascites du to BCS in a 28-year old patient some years ago before the seminal discovery of Jak 2 mutation.^6^

Standard clinical and laboratory analyses including cytomorphological examination of bone marrow, thrombophilic screening, ultrasound Doppler investigation of portal vessels were performed. The major hereditary genetic defects favoring thrombosis (AT-III, Protein C, Protein S, and Protein C resistance) were excluded, however an heterozygous mutations of methylenetetrahydrofolatreductase C677T and Plasminogen Activator Inhibitor-1 (PAI-1) were found. Level of homocysteine was also increased up to 24 mcmol/L(normal level 12 mcmol/L). On the basis of the above data a thrombophilic state was suspected. No hematological disorder was recognized. The patients was started on anticoagulant therapy obtaining an improvement of ascites.

Only 4 years after the disease beginning a diagnosis of chronic MPN (primary myelofibrosis) was established according to WHO criteria: multilineage hyperplasia with megacaryocyte dysplasia and myelofibrosis in the bone marrow histology, palpable splenomegaly, increased serum LDH, anemia, detection of the JAK2 V617F positive mutation in absence of cytogenetic abnormalities, the hyperhomocysteinaemia was confirmed and considered an additional risk factor of thrombosis. Only after verification of the diagnosis anticoagulant and cytostatic therapy was started. The result was positive: we observed a resolution of clinical symptoms, normalization of blood counts and partial restore of blood flow in hepatic veins. But self discontinuation of the antithrombotic therapy by patient without informing her doctors provoked rethrombosis in the same localization. The resumption of anticoagulant therapy led to positive clinical effect again. We observed disappearance of ascites, relief of pain, normalization of body temperature, reduction of liver sizes, recanalization of two hepatic veins. The general state of patient’s health improved. The described clinical case shows that the BCS development can be the first sign of myeloproliferative neoplasm.

Hereditary thrombophilia factors could be very important in maintaining the thrombotic state in patients with chronic MPN. Therefore special attention should be paid not only to cytoreductive therapy for normalization of blood counts, but also to perpetual intake of anticoagulants.
